# Supplementary material for: Currency risk analysis of belt and road initiative countries
Source: PLoS One. 2024 Jul 12;19(7):e0307209. doi: 10.1371/journal.pone.0307209 (PMC11244813; doi:10.1371/journal.pone.0307209)
Supplement: Attachment — Submitted filename: Response to Reviewers.docx [file pone.0307209.s002.docx]

**Response to Editor’s comments**

**Comment 1.** Please ensure that your manuscript meets PLOS ONE's style requirements, including those for file naming. The PLOS ONE style templates can be found at

https://journals.plos.org/plosone/s/file?id=wjVg/PLOSOne_formatting_sample_main_body.pdf and

https://journals.plos.org/plosone/s/file?id=ba62/PLOSOne_formatting_sample_title_authors_affiliations.pdf

**Response**: We have carefully reviewed PLOS ONE's style requirements, including those for file naming, and have made the necessary adjustments. Our revised manuscript now adheres to all the specified guidelines.

**Comment 2.** Thank you for stating the following financial disclosure:

“the State Key Laboratory of Desert and Oasis Ecology: Xinjiang Institute of Ecology and Geography, CAS (E1510107)”

Please state what role the funders took in the study.  If the funders had no role, please state: "The funders had no role in study design, data collection and analysis, decision to publish, or preparation of the manuscript."

If this statement is not correct you must amend it as needed.

Please include this amended Role of Funder statement in your cover letter; we will change the online submission form on your behalf.

**Response**: Regarding the financial disclosure provided, we acknowledge the support of the State Key Laboratory of Desert and Oasis Ecology: Xinjiang Institute of Ecology and Geography, CAS. In accordance with your request, we confirm that the funders played no role in the study design, data collection and analysis, decision to publish, or preparation of the manuscript.

**Comment 3.** We note that Figures 3,7,8 and 10 in your submission contain [map/satellite] images which may be copyrighted. All PLOS content is published under the Creative Commons Attribution License (CC BY 4.0), which means that the manuscript, images, and Supporting Information files will be freely available online, and any third party is permitted to access, download, copy, distribute, and use these materials in any way, even commercially, with proper attribution. For these reasons, we cannot publish previously copyrighted maps or satellite images created using proprietary data, such as Google software (Google Maps, Street View, and Earth). For more information, see our copyright guidelines: <http://journals.plos.org/plosone/s/licenses-and-copyright.>

We require you to either (1) present written permission from the copyright holder to publish these figures specifically under the CC BY 4.0 license, or (2) remove the figures from your submission.

**Response**: We have addressed the issue raised by the editor and reviewer by replacing Figures 3, 7, 8, and 10 with new figures. The original figures were based on standard map production with the map approval number GS(2016)1667 from the Chinese Ministry of Natural Resources (http://bzdt.ch.mnr.gov.cn/), and the basemap remains unaltered. We have also improved our result displays and discussions as suggested by Reviewer #2, who pointed out the shortcomings of the original figures in making a comparison between five currencies. In response to this feedback, we have tried another way to display our results and supporting data for our discussion. We have grouped the results from 142 countries by a new method that labels them according to their currency competition status and further discuss these groups, thereby improving our paper.

**Comment 4.** We are unable to open your Supporting Information file [PlosoneData.7z]. Please kindly revise as necessary and re-upload.

**Response**: We have re-uploaded our data file. We hope that it can be opened successfully this time.

**Response to reviewer’s comments**

**Reviewer #1**

**Comment 1.** I found this study very interesting as it provides valuable insights into currency risk analysis in Belt and Road Initiative countries. Nonetheless, I suggest authors consider the following comments to significantly improve the overall quality of their manuscript.

**Response**: Thank you for your valuable feedback. We appreciate your positive comments on the study and are grateful for your suggestions to improve the overall quality of our manuscript. We will carefully considered and addressed the comments you have provided to enhance the value and impact of our research.

**Comment 2.** Authors should add a section on the literature review establishing the theoretical context and describing the results of previous studies on the subject.

**Response**: Thank you for your feedback. We have addressed your suggestion by including a section on the literature review to establish the theoretical context and describe the results of previous studies on the subject. Specifically, we have added three sections on the literature review, covering debt sustainability issues, currency risk, and currency competition.

The literature review on debt sustainability issues explores the existing research on debtors’ solvency and economic stability, while also highlighting the significance of currency risk. This can be found in lines 51-72 of the manuscript.

The literature review on currency risk is organized into two dimensions and can be found in lines 73-106.

Additionally, we have included a section on the currency competition theory as a new entry point for our research, which can be found in lines 107-140.

We believe that these additions provide a comprehensive theoretical context and an overview of previous studies on the subject. Thank you for your valuable input.

**Comment 3.** You must provide references or justification supporting each of the thirteen variables involved in the construction of the CCI index (see table 1) and each equation/model.

**Response**: Included references for each variable used in our CCI index, and the modifications made can be found in lines 161-183 and 212-216.

**Comment 4.** When discussing the results, add additional references and stylized facts that explain/justify the results. You should also compare your results to those of previous studies.

**Response**: Additional references and stylized facts have been incorporated into the Discussion section to further explain and justify the results. The characteristics of currency competitiveness, bilateral trade, and bilateral investment are summarized using the data, and the results are compared with previous studies.

We have added facts about the characteristics of CCI before and after BRI and compared them with the literature (Lines 443-456).

The characteristics of CCI at present and in future expectations have been included, aligning with the literature (Lines 474-477).

A comparison of the results of this paper with the literature in the continental groupings has been added (Lines 544-549).

We have included a comparison between the outcomes of this study and those in the existing literature, demonstrating similar trends but with more detailed findings (Lines 599-609).

**Comment 5.** The conclusion should also present the detailed policy and practical implications of your findings and highlight the limitations of the study and avenues of research for future analyses.

**Response**: The conclusions have been revised to address the specific concerns raised by the reviewer. We have provided more detailed policy and practical implications of our findings, as well as highlighted the limitations of the study and avenues for future research. Specifically, we have revised the presentation of results in the conclusions to provide more detail on the research results (Lines 613-633), added policy recommendations and discussed the marginal contribution of the paper (Lines 634-641), and included content on limitations and future research directions (Lines 642-647). Thank you for the valuable feedback, and we believe these revisions have strengthened the conclusions of the paper.

**Reviewer #2**

**Comment 1.** The relevance of the topic is well presented. It deals with an interesting, timely and economically beneficial topic, but it needs significant development.

**Response**: Thank you for your feedback. I appreciate your recognition of the relevance of the topic. I I have provided a more comprehensive and in-depth analysis.

**Comment 2.** The literature review is very superficial, only tangentially referring to the debates and methodologies, not presenting the research niche in depth. What is missing from the literature review is a more detailed description of the other methodologies that have been used to research the topic. From this, it could be deduced why the author uses this methodology to answer his research question. I miss the operationalisation derived from the theoretical framework. I would suggest a more in-depth presentation of the theoretical background that underpins this methodology. It refers to different models (e.g. Anchor Effect of Currency), but we do not know in depth what those models say. In the theoretical introduction, the models used and their main findings should be discussed, so that we can later better understand the results that are compared with them.

**Response**: Thank you for your suggestion. We have supplemented the literature review in the introduction with three sections, debt sustainability issues, currency risk, and currency competition. Lines 51-72 provide a compendium of literature perspectives on debt sustainability issues, lines 73-106 introduce the content of the literature studies on currency risk, and lines 107-140 review the literature on currency competition used in this paper.

In addition, we have added a brief introduction to the Anchor Effect of Currency to the literature review in lines 125-127, which is described in detail in the Methods section in lines 178-211. We believe that these additions address the concerns raised and provide a more in-depth presentation of the theoretical background that underpins the methodology used in this research.

**Comment 3.** From row 92, the description of the indicators should also be provided in Table 1. There is no detailed description that would justify a textual description. The content of Table 2 (data sources) should be included in Table 1, two tables are unnecessary. Can you provide data on the original database, the number of unestimated observations per variable? Give us a better insight into the database.

**Response**: Revised Tables 1 and 2 to address the reviewer’ s comments. The description of the indicators has been added to Table 1, and the content of Table 2 (data sources) has been included in Table 1 as well. This eliminates the need for two separate tables. We have also provided citations for the 13 indicators in the textual descriptions.

Additionally, we have updated Table 2 to include information on the original database, as well as the number of unestimated observations per variable. This provides a better insight into the database.

**Comment 4.** Improve the graphical appearance of the charts, they are now very undemanding. Figures 3, 7, and 10 are not suitable for comparison in their present form. This can be submitted as an appendix, but I would suggest that the authors cluster the countries based on the results. It would be much better to illustrate their results. In the text, they could explain the reasons behind the clusters.

**Response**: We have improved the graphical appearance of the charts by removing the map displays for Figures 3, 7, and 10, and made changes to the results display. We have presented the results in both temporal and spatial dimensions. Under the spatial dimension, we have modified the results display by showing the Top 20 CCI countries by currency and continent of the 4th quarter of 2020 (Table 5), and the CCI distribution in BRI countries in the 4th quarter of 2020 ranked from highest to lowest for each currency (Fig 2), paving the way for the grouping by currency competition status in section 4.2.2.

In the Discussion, we have grouped the results of the study CCI in a spatial perspective, retaining the original continent grouping and adding a new grouping on the state of currency competition. We have also added new discussion based on this grouping. We believe these changes have significantly improved the graphical appearance of the charts and the overall presentation of the results.

**Comment 5.** In line 303, the author refers to "discussions on currency competitiveness", but we do not find anything about them in the theoretical background.

**Response**: Added a section on currency competition and competitiveness in the literature review of the introduction, specifically in lines 107-140. Thank you for bringing this to our attention.

**Comment 6.** The conclusion is very brief. More detail should be given on the results of this research, what it adds to the literature, what its limitations are, and what other new research directions the authors have discovered during their research. What region-specific policy recommendations can they make based on their findings.

**Response**: We have revised the conclusion to provide more detail on the research results, specifically in lines 613-633. We have included region-specific policy recommendations and highlighted the paper’s contribution based on the findings in lines 634-641. Additionally, we have added content on the limitations of the study and future research directions in lines 642-647.
