# Supplementary material for: Currency risk analysis of belt and road initiative countries
Source: PLoS One. 2024 Jul 12;19(7):e0307209. doi: 10.1371/journal.pone.0307209 (PMC11244813; doi:10.1371/journal.pone.0307209)
Supplement: Attachment — Submitted filename: Response to Reviewers.docx [file pone.0307209.s003.docx]

**Response to Editor’s comments**

**Comment 1.**

ACADEMIC EDITOR:

The quality of the English needs to be improved. Therefore, I request that you consult professional English-editing services (e.g., Editage, Enago, etc.) and obtain an English-editing certificate from the service provider to be submitted along with the edited version of this manuscript.

**Response**: Thank you for your feedback. We have already engaged professional English-editing service (LetPub) to improve the quality of the manuscript. We have obtained an English-editing certificate from the service provider and will submit it along with the edited version of the manuscript.

**Response to reviewer’s comments**

**Reviewer #1 and #2**

**Comment 1.** All comments have been addressed.

**Response**: Thank you for your feedback. We have carefully addressed all comments and made the necessary revisions. We appreciate your time and effort in reviewing our work.
